# CLSM-Guided Imaging to Visualize the Depth of Effective Disinfection in Endodontics

**DOI:** 10.3390/antibiotics14121201

**Published:** 2025-12-01

**Authors:** Rebecca Mattern, Sarah Böcher, Gerhard Müller-Newen, Georg Conrads, Johannes-Simon Wenzler, Andreas Braun

**Affiliations:** 1 Department of Operative Dentistry, Periodontology, and Preventive Dentistry, Rheinisch-Westfälische Technische Hochschule (RWTH) University Hospital, Pauwelsstrasse 30, 52074 Aachen, Germany; 2 Institute of Biochemistry and Molecular Biology, Rheinisch-Westfälische Technische Hochschule (RWTH) University Hospital, Pauwelsstrasse 30, 52074 Aachen, Germany; 3 Division of Oral Microbiology and Immunology, Department of Operative Dentistry, Periodontology, and Preventive Dentistry, Rheinisch-Westfälische Technische Hochschule (RWTH) University Hospital, Pauwelsstrasse 30, 52074 Aachen, Germany

**Keywords:** endodontic treatment, root canal disinfection, *Enterococcus faecalis*, CLSM, LIVE/DEAD staining, irrigation depth, sodium hypochlorite, EDTA, EDDY

## Abstract

Background/Objectives: Important goals of endodontic treatment procedures are to effectively eliminate microorganisms from the root canal system and prevent reinfection. Despite advances in techniques, these goals continue to be difficult to achieve due to the complex anatomy of the root canal system and bacterial invasion into the dentinal tubules of the surrounding root dentin. This pilot study aimed to refine a confocal laser scanning microscopy (CLSM) model with LIVE/DEAD staining to quantitatively assess the depth of effective disinfection by endodontic disinfection measures. Methods: Thirty caries-free human teeth underwent standardized chemo-mechanical root canal preparation and were inoculated with *Enterococcus faecalis*. Following treatment, CLSM-guided imaging with LIVE/DEAD staining allowed for differentiation between vital and dead bacteria and quantification of the depth of effective disinfection. Results: An average depth of bacterial eradication of 450 µm for conventional and 520 µm for sonically activated irrigation (EDDY) could be observed with significant differences (*p* < 0.05) in the coronal and medial positions. Conclusions: The results indicated that sonically activated irrigation (EDDY) provided a more homogeneous (omnidirectional) irrigation pattern compared to conventional irrigation. The study highlights the importance of effective disinfection strategies in endodontics, emphasizing the need for further research on the depth of effective disinfection of endodontic disinfection measures and the optimization of disinfection protocols.

## 1. Introduction

Important goals of endodontic treatment procedures are to effectively eliminate microorganisms from the root canal system by means of thorough and carefully performed chemo-mechanical treatment [1,2] and to prevent reinfection [3]. Despite many advances in modern endodontics, these goals continue to be difficult to achieve due to the complex anatomy of the root canal system, including lateral or accessory canals, isthmuses, anastomoses, apical deltas, and irregular canal configurations [4,5]. In addition, bacteria can penetrate the dentinal tubules of the surrounding root dentin, making them even more difficult to eliminate [5,6]. Thorough disinfection is crucial for long-term treatment success, since the persistence of microorganisms is one of the main reasons for endodontic treatment failure [4,7].

Mechanical cleaning of the root canal system is restricted, with more than one-third of the canal surface remaining untouched by endodontic instruments [8,9,10]. Irrigation therefore plays a decisive role during endodontic treatment, as it allows for chemical dissolution of tissue residues, the removal of debris and the smear layer, and the detachment of biofilm [3,10]. Sodium hypochlorite (NaOCl) is the disinfectant most commonly used in endodontics, due to its antimicrobial efficacy and tissue-dissolving properties [5,11]. However, its penetration depth into the surrounding dentin is limited. Various studies have shown that difficult-to-treat Gram-positive bacteria such as *Enterococcus faecalis* are able to colonize the dentinal tubules to a depth of up to 1350 µm [12,13,14,15,16], while irrigation solutions such as sodium hypochlorite can only penetrate the dentinal tubules to a much shallower depth [14,15,17,18,19,20]. Residual bacteria may then lead to reinfection, with *E. faecalis* in particular being linked to cases of endodontic treatment failure [7,21,22,23,24,25].

Various techniques for activating irrigation solutions have been proposed in order to improve the penetration depth of irrigation solutions into the surrounding root dentin, including sonically activated irrigation methods [10]. Endodontic disinfection measures have been the subject of extensive research for many years, and numerous protocols for evaluating and quantifying the depth of penetration into the surrounding root dentin have been proposed and investigated, including microbial cultivation and histological methods [15,26], dye penetration and discoloration tests [10,19], and scanning electron microscopy (SEM) imaging [14]. To date, only a few studies have used confocal laser scanning microscopy (CLSM) and functional staining.

The aim of this pilot study was to fine-tune an experimental model using CLSM-guided imaging and LIVE/DEAD staining previously established by our research group [27], allowing for clear differentiation between vital and dead bacterial cells. The model was then evaluated for its ability to quantify the depth of bacterial colonization as well as the depth of effective disinfection of endodontic disinfection measures, comparing conventional irrigation and an adjunctive therapy method.

## 2. Results

**Figure 1:**
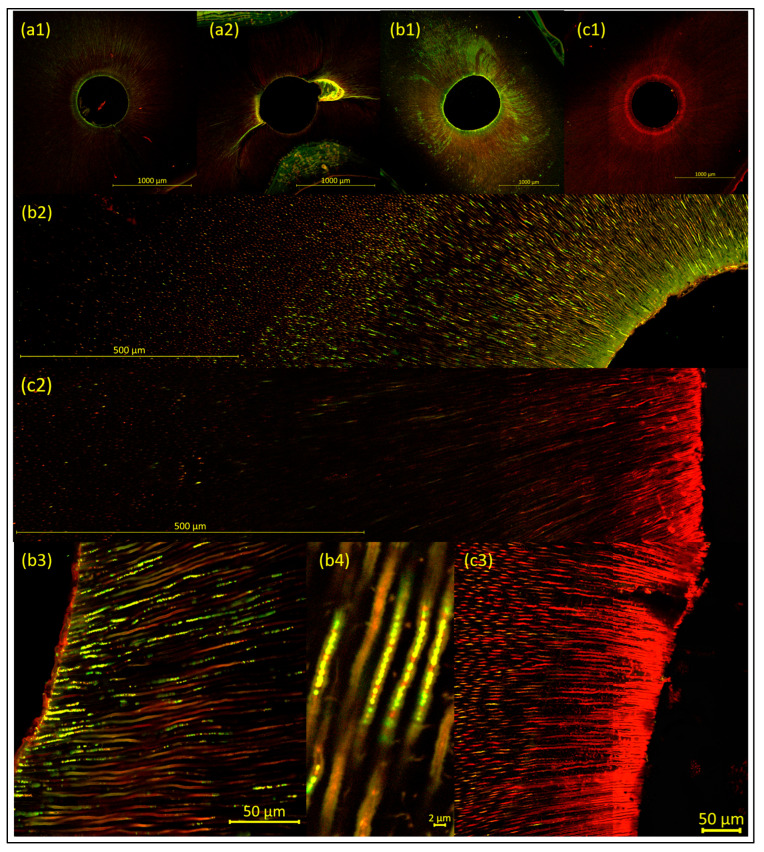
Representative images of the samples, to illustrate the methodology (visualized using CLSM and LIVE/DEAD staining, with increasing magnification from 1 to 4). Images (**a1**,**b1**,**c1**) show overviews, with a 10× objective lens; the root canal wall appears as a distinct fluorescent line. Images (**b2**,**c2**) are 9 × 2 tile scans with a 40× objective lens. Images (**b3**,**b4**,**c3**) are detailed images of dentinal tubules, with a 40× objective lens. (**a1**,**a2**) The negative control group, with (**a1**) showing the ideal result of a sample with neither bacteria nor released DNA/RNA remaining, with complete mechanical instrumentation of the root canal and open, clear, and debris-free dentinal tubules. In contrast, (**a2**) shows the consequences of incomplete mechanical instrumentation of the root canal, with a biofilm/debris-rich lateral canal remaining. (**b1**–**b4**). The positive control group after microbial inoculation (without treatment—i.e., disinfection measures). The dentinal tubules show bacterial cell–related green fluorescence, originating from vital biofilm and/or single bacterial cells. (**c1**–**c3**). The sonically activated irrigation (EDDY) group after treatment. The samples show dead bacterial cells (red fluorescence) with vital bacterial cells (green fluorescence) remaining in deeper dentin layers. Additional red background fluorescence is clearly visible and needs to be carefully distinguished from red fluorescence arising from dead bacteria and/or released DNA/RNA.

Before bacterial inoculation, it had to be ensured that neither bacteria nor DNA/RNA remained after autoclaving of the samples. Mechanical instrumentation of the root canal had to be complete, and after pretreatment (Pr2, without NaOCl), the orifices of the dentinal tubules had to be open, clear, and free of debris, with no residual smear layer (negative control group). The ideal result is shown in Figure 1(a1), while Figure 1(a2) shows the consequences of incomplete mechanical preparation of the root canal, with a biofilm/debris-rich lateral canal remaining. In contrast to the parts of the root canal that were accessible to mechanical instrumentation, the biofilm/debris-rich side canal showed increased fluorescence, originating from remnants both of odontoblasts/pulp tissue and microorganisms.

After 4 weeks of bacterial inoculation (without treatment—i.e., disinfection measures [positive control group]), CLSM images were obtained from the positive control group to ensure the validity of the experimental model. In all samples, bacterial cell–related green fluorescence was detected in the dentinal tubules, originating from vital biofilm and/or single bacterial cells, and biofilm-like structures were observed on the root canal walls (Figure 1(b1–b4)).

After bacterial inoculation and subsequent treatment (i.e., disinfection measures [test groups]), the samples showed dead bacterial cells (red fluorescence) with vital bacterial cells (green fluorescence) remaining in deeper dentin layers (Figure 1(c1–c3)). Almost no bacteria remained on the root canal walls, except for a few irregular, shrunken, and damaged bacterial cells.

### 2.1. Evaluation of Bacterial Colonization

The greatest invasion depth for *E. faecalis* (bacterial colonization) was observed to be 2342 µm into the root dentin (mean penetration depth 480 µm), with the penetration depth decreasing from sclerosis grade (SCG) 1 to 3 and from coronal to apical [27]. In addition, the penetration depth was significantly greater in the orovestibular than in the mesiodistal direction, which could be explained by the butterfly pattern associated with increasing degrees of sclerosis [27].

It should be noted that in the original literature, sclerosed mesiodistal dentin areas appeared significantly whiter in cross-sections and were therefore described as butterfly-shaped [28,29]; this is referred to as the “butterfly pattern” in the present study. In contrast, when green-red LIVE/DEAD staining is used, the orovestibular areas, which have more open tubules with a greater depth of bacterial invasion and bacterial eradication, appear conspicuously butterfly-shaped due to the increased fluorescence of living/dead bacterial cells; this is referred to as the “butterfly penetration pattern” in the present study.

### 2.2. Evaluation of the Efficacy of Disinfection Measures (Bacterial Eradication)

The mean penetration depth of the disinfection measures and thus the depth of bacterial eradication was 450 µm into the root dentin with conventional irrigation and 520 µm for sonically activated irrigation (EDDY). The greatest penetration depth of 2243 µm was achieved for sonically activated irrigation (EDDY) in SCG 1 samples in a coronal position (Table 1).

**Figure 2:**
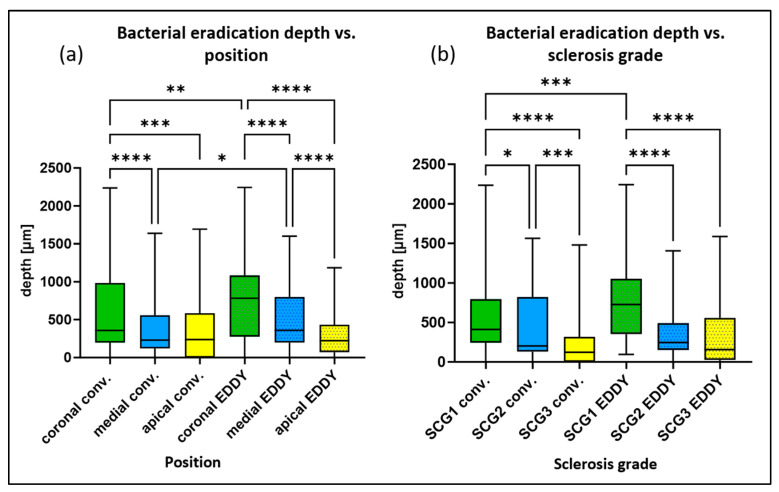
Box plots for the depth of bacterial eradication (mean values of the 16 individual measurements per sample) depending on two parameters: (**a**) position (coronal > medial > apical) and (**b**) degree of sclerosis (SCG 1 > SCG 2 > SCG 3) for the groups with conventional and sonically activated irrigation (EDDY). * *p* < 0.05, ** *p* < 0.01, *** *p* < 0.001, **** *p* < 0.0001.

The depth of bacterial eradication was dependent on position (coronal > medial > apical). In the conventional irrigation group, statistically significantly (*p* < 0.05) higher values were observed for the coronal position, with a mean penetration depth of 600 µm in comparison with the medial position, with a mean penetration depth of 360 µm and with the apical position, with a mean penetration depth of 390 µm. In the group with sonically activated irrigation (EDDY), the mean depths of bacterial eradication were 760 µm in the coronal position, 520 µm in the medial position, and 280 µm in the apical position, with statistically significant (*p* < 0.05) differences between all positions. In addition, sonically activated irrigation (EDDY) showed statistically significantly (*p* < 0.05) greater penetration depths in the coronal and medial positions in comparison with conventional irrigation (Figure 2a).

As discussed in our previous report [27], the depth of bacterial eradication was also dependent on the degree of sclerosis (SCG), decreasing from SCG 1 to SCG 3 (SCG 1 > SCG 2 > SCG 3). For conventional irrigation, statistically significant differences (*p* < 0.05) were observed between all groups, with a mean depth of bacterial eradication of 590 µm for SCG 1, 440 µm for SCG 2, and 230 µm for SCG 3. For sonically activated irrigation (EDDY), statistically significant differences (*p* < 0.05) were found between SCG 1, with a mean depth of bacterial eradication of 760 µm, compared with SCG 2, with a mean depth of 360 µm and SCG 3, with a mean depth of 360 µm (Figure 2b). Statistically significant differences (*p* < 0.05) between the two disinfection measures were only observed for SCG 1 samples (all positions), with sonically activated irrigation (EDDY) showing greater penetration depths.

**Figure 3:**
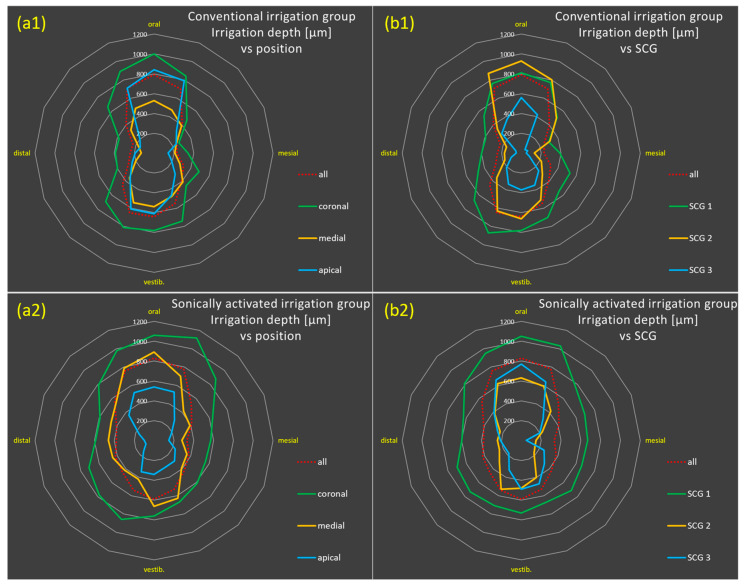
Radar charts for bacterial eradication based on the 16 individual penetration depth values (16 position-dependent mean values, in µm) for the groups with conventional irrigation (**a1**,**b1**) and with sonically activated irrigation (EDDY) (**a2**,**b2**) relative to position (coronal to apical, (**a**)) and degree of sclerosis (SCG 1–3, (**b**)). The diagrams show deeper penetration depths in the orovestibular than in the mesiodistal direction (butterfly penetration pattern) in both groups, although the effect is more pronounced in the conventional irrigation group, while sonically activated irrigation (EDDY) resulted in a more homogeneous radial (omnidirectional) irrigation pattern.

Figure 3 illustrates the depth of bacterial eradication based on the 16 individual penetration depth values (16 position-dependent mean values). Greater penetration depths in the orovestibular direction than in the mesiodistal direction (butterfly penetration pattern) are clearly visible in both groups, although the effect is more pronounced in the conventional irrigation group. Thus, sonically activated irrigation (EDDY) resulted in a more homogeneous radial (omnidirectional) irrigation pattern than conventional irrigation.

## 3. Discussion

*E. faecalis*, well known for its involvement in recurrent infections, is reported to invade deep into the dentinal tubules [30,31]. It was therefore chosen as the test organism here. Various studies have shown that severe infection/colonization with *E. faecalis* occurs up to 400–500 µm from the root canal lumen and that individual bacteria can reach invasion depths of up to 1000–1350 µm [12,13,14,15,16]. In the present study, individual vital bacterial cells (*E. faecalis*) were even detected at a depth of 2342 µm. However, it should be noted that the artificial inoculation model (A-Pr2) used in the present study might be the reason for such deep colonization [27], which may differ from the bacterial colonization observed in clinical situations.

As previous research showed that irrigation solutions are only able to penetrate the dentinal tubules to a much shallower depth [14,15,17,18,19,20], various adjuvant therapy methods have been proposed in efforts to improve the penetration depth of disinfection measures, including irrigation methods with ultrasonic or sonic activation, hydrodynamic irrigation methods, and laser-based therapy options. In the present study, activation of the irrigation solution by means of EDDY (VDW GmbH, Munich, Germany), a sonically activated irrigation system with frequencies of 5000–6000 Hz, was investigated as an adjunctive treatment method. Other devices, such as the EndoActivator (Dentsply Sirona, Bensheim, Germany), have also been classed and investigated as sonically activated irrigation systems, although they operate at lower frequencies of 2000–3000 Hz, making comparison difficult. Ultrasonically activated irrigation systems were not evaluated in the present study, although limited evidence suggests that sonically activated irrigation systems (EDDY) show comparable results regarding effects within the root canal itself [32]. It is also important to point out that the present study focused on evaluating the penetration depth (or, more precisely, the depth of effective disinfection) of disinfection measures into the surrounding root dentin. Effects within the root canal itself (e.g., debris removal or tissue dissolution) or side effects affecting surrounding tissue (e.g., apical extrusion) were not addressed in this study and were therefore not evaluated.

Various methods of assessing and quantifying the depth of penetration of endodontic disinfection measures into the surrounding root dentin have been evaluated in recent years. Early studies investigating the effects of disinfection measures and root canal medications used microbial culture methods [12,26]. A study by Buck et al. (2001) investigated the penetration depth of endodontic disinfection measures by gradually drilling into the surrounding dentin (starting from the root surface towards the root canal) of teeth infected with *E. faecalis* and culturing the shavings to examine for surviving bacteria in the outer, middle, and pulpal thirds of the root dentin [26]. A study by Haapasalo et al. (1987) used dentin blocks from bovine teeth that had been inoculated with *E. faecalis*. To evaluate the depth of the disinfecting effect of root canal medication into the surrounding dentin, zones 100 µm thick were gradually removed from the root canal wall using a drill and cultured to determine the number of surviving bacteria [12]. More recent studies by Nourzadeh et al. (2017) and Parolia et al. (2021) used a similar approach, in which dentinal layers were gradually removed from the inner surface of the root canal, followed by culturing. The authors found that NaOCl had some antibacterial activity at all depths investigated—200 and 400 µm [33,34] as well as 600 µm [34]—decreasing with increasing depth.

Another study on the penetration depth of irrigation solutions used histological methods and observed a zone without tubular infection averaging 130 µm with NaOCl [15].

Later studies on the penetration depth of endodontic disinfection measures used dye penetration tests, in which a dye was added to the irrigation solutions to visualize the penetration depth into the surrounding dentin, or the dye itself was used as a final irrigation solution and activated by means of various adjuvant disinfection measures (e.g., sonically or ultrasonically activated irrigation procedures). The dyes used included acid fuchsin [35], alizarin red [36], Chinese ink [37], rhodamine B [17], Patent Blue V [38] or, in later studies, mainly methylene blue [10,39,40]. The penetration depth was assessed using categories or scores [35,36], the percentage of the stained area relative to the total area of the root dentin [37,38], or based on the penetration depth of the dye measured from the root canal wall [10,17,39,40]. Due to the different dyes used and the varying assessment of the results, comparison is extremely difficult. Paragliola et al. (2010), for example, reported a statistically significant (*p* < 0.05) difference between the control group (NaOCl without activation) and sonically activated irrigation (EndoActivator) when all samples (in coronal, medial, and apical positions) were analyzed together; no statistically significant differences (*p* > 0.05) were found when the different positions were considered individually [36]. Macías et al. (2018) investigated the percentage of the stained area in relation to the total area of the root dentin and did not find any statistically significant differences (*p* > 0.05) between passive irrigation (all positions 9.19%; coronal 11.9%; medial 10.4%; apical 5.0%) and sonically activated irrigation (EndoActivator; all positions 9.42%; coronal 11.4%; medial 12.8%; apical 4.1%) [37]. Studies evaluating the metric penetration depth into the surrounding dentin showed varying results. For example, Rajakumaran et al. (2019) used a dye penetration test with rhodamine B and reported penetration depths of 155 µm (coronal), 91 µm (medial), and 30 µm (apical) with conventional irrigation [17]. The following studies using methylene blue have described considerably better results. Galler et al. (2019) observed penetration depths of 775 µm for manual dynamic activation and 985 µm for sonically activated irrigation (EDDY) [10], while Widbiller et al. (2023) noted penetration depths of 616 µm for conventional irrigation and 1131 µm (coronal 1205 µm; medial 1139 µm; apical 1050 µm) for sonically activated irrigation (EDDY) [40]. Dadhich et al. (2023) reported penetration depths of 594 µm for irrigation without activation, 649 µm for manual dynamic activation, and 2277 µm for sonically activated irrigation (EndoActivator) [39].

Important and frequently discussed criticisms of dye penetration tests are that the addition of the dye may impair the properties of the irrigation solutions, or that the dye molecules penetrate independently of the irrigation solution itself (more or less deeply—since the dyes may have a different molecular size than the irrigation solutions, which can lead to different penetration properties) and thus “visualize” incorrect penetration depths [18]. Another consideration is that the maximum penetration depth of the irrigation solutions does not necessarily correspond to the actual depth of effective disinfection. Although the dye marks the actual penetration depth of the irrigation solution, the concentration of the irrigation solution may no longer be sufficient for effective disinfection in deeper dentin layers, meaning that no (or only a reduced) antibacterial efficacy can be achieved there [18]. Conversely, the dye molecules may be altered by the strong oxidizing activity of NaOCl, causing the staining properties to be lost or altered [18]. The choice of dye used also appears to have an influence on the penetration depth. Studies using methylene blue, for example, have consistently reported greater penetration depths than studies using rhodamine B. Due to the different molecular sizes (methylene blue 320 g/mol [41], rhodamine B 479 g/mol [42]), hydrophilicity, and interaction with the dentin matrix, the choice of dye for penetration and diffusion studies in dental tissues appears to be of crucial importance. Another point of criticism is that the process of cutting the tooth samples may lead to further distribution of the dye (blurring) in the dentin, which would result in overestimation of the actual penetration depth [43].

To overcome these disadvantages of dye penetration tests, discoloration tests were later introduced. In these, the penetration depth into the root dentin was evaluated on the basis of the ability of NaOCl-based irrigation solutions to bleach dentin/dentinal tubules previously stained with copper sulfate solutions [18] or crystal violet [19,20,44,45]. The penetration depth was then determined by evaluating the area of the bleached root dentin [18] and/or the depth of the bleached area measured from the root canal wall [19,20,44,45]. Giardino et al. (2017) used a discoloration test with a copper sulfate solution and measured average penetration depths of 39 µm (coronal), 46 µm (medial), and 45 µm (apical) for NaOCl [18]. Studies using crystal violet showed different results. Faria et al. (2019) observed penetration depths of 47.6–118.4 µm for NaOCl [19], while Zou et al. (2010) noted penetration depths ranging from 77 to 300 µm depending on time, concentration, and temperature [45]. A study by Virdee et al. (2020) compared conventional irrigation (coronal 179–272 µm, medial 158–259 µm, apical 38–171 µm) to manual dynamic activation (coronal 240–379 µm, medial 237–411 µm, apical 159–232 µm) and sonically activated irrigation (EndoActivator; coronal 280–329 µm, medial 275–310 µm, apical 121–165 µm) and found statistically significant (*p* < 0.05) differences for the coronal and medial positions (conventional < manual < sonic). For the apical position, statistically significantly (*p* < 0.05) greater penetration depths were observed for manual dynamic activation (conventional < sonic < manual). As with dye penetration tests, discoloration tests are also controversial, because the depth of discoloration does not necessarily correspond to the penetration depth of the irrigation solution or the actual depth of effective disinfection [20].

Other studies, such as Cheng et al. (2016), used SEM imaging and cell count methods and observed intact *E. faecalis* cells at a depth of 200 µm after irrigation with NaOCl [14]. One disadvantage of SEM imaging is that it does not provide any information about the viability of the bacteria, whereas CLSM imaging in combination with LIVE/DEAD staining makes it possible to distinguish between vital and dead bacterial cells and thus allows for assessment of the actual depth of effective disinfection [27].

To date, only a few studies have used CLSM imaging and LIVE/DEAD staining [13,46,47,48], and metric analyses on the penetration depth of different disinfection measures have rarely been performed. Al Shahrani et al. (2014), for example, reported that after irrigation with NaOCl, the red fluorescence of dead bacteria was only visible on the surface layer, while deeper layers showed green fluorescence of vital bacterial cells. However, CLSM imaging was only used for illustrative purposes and quantitative analysis was not performed [48]. A study by Neelakantan et al. (2015) also did not evaluate the exact penetration depth or the depth of effective disinfection but demonstrated a certain antibacterial efficacy of NaOCl even at a depth of 400 µm [47], while Zeng et al. (2018) noted a certain efficacy at a depth of 150 μm [46]. Only one study, by Vatkar et al. (2016), measured the depth achieved by disinfection measures and observed a zone of dead bacteria to a depth of 88–110 µm with NaOCl [13].

The results of the present study show that conventional irrigation achieved a mean penetration depth of 450 µm into the root dentin (coronal 600 µm, medial 360 µm, apical 390 µm), while sonically activated irrigation (EDDY) achieved a mean penetration depth of 520 µm (coronal 760 µm, medial 520 µm, apical 280 µm). Thus, the penetration depths for conventional irrigation measured in the present study are greater than the results reported from studies that used histological methods, SEM, dye penetration tests with rhodamine B, or discoloration tests that reported penetration depths of approximately 38–272 µm with conventional irrigation [14,15,17,18,19,20]; but they are less than the penetration depths determined in studies using color penetration tests with methylene blue (approximately 616–775 µm) [10,39,40]. The results of studies using CLSM and LIVE/DEAD staining vary from lower (88–110 µm [13]) to similar, as Neelakantan et al. (2015) were able to demonstrate at least a slight antibacterial efficacy for conventional irrigation with NaOCl at a depth of 400 µm [47]. Similarly, results from recent microbial culture studies have shown a certain degree of antibacterial efficacy at 400 µm [33] and 600 µm [34].

Only a few studies have investigated sonically activated irrigation, including studies that used dye penetration tests with methylene blue. Penetration depths of 985 µm [10] and 1131 µm [40] were reported for EDDY and 2277 µm for the EndoActivator [39], with the results being significantly better than the penetration depths determined in the present study. Another study using discoloration tests with crystal violet reported penetration depths of 121–329 µm with the EndoActivator [20], which are lower than the results of the present study.

In the present study, statistically significantly (*p* < 0.05) greater penetration depths were noted for sonically activated irrigation (EDDY) in the coronal and medial positions, a finding that is in line with some previous studies in which statistically significantly better (*p* < 0.05) penetration depths were observed with sonically activated irrigation (EndoActivator) in comparison with conventional needle irrigation and manual dynamic activation [20,39]. In contrast, other studies did not report any statistically significant (*p* > 0.05) differences between conventional [10] and manual dynamic activation [40] and sonically activated irrigation (EDDY) [10,40]. Interestingly, in the present study, sonically activated irrigation (EDDY) was associated with smaller penetration depths in the apical position in comparison with conventional irrigation, an observation that is again consistent with some previous findings [20,37], while Galler et al. (2019) reported higher penetration depths with sonically activated irrigation (EDDY) even in the apical position [10]. In the present study, a statistically significant (*p* < 0.05) difference was found between conventional and sonically activated irrigation (EDDY) in the coronal and medial positions, with EDDY showing higher values, but not in the apical position. One possible explanation for this might be a low volume of disinfectant solution available next to the polymer tip and a limited oscillation amplitude due to the limited space in the apical region.

Although both groups showed greater penetration depths in the orovestibular than in the mesiodistal direction (butterfly penetration pattern), which has been described as a “barbell shape” in earlier studies [38], this effect was more pronounced in the conventional irrigation group, while sonically activated irrigation (EDDY) resulted in a more homogeneous radial (omnidirectional) irrigation pattern.

Differences in the findings of the various studies may therefore also result from differences in the sectioning of the samples. Many studies have used longitudinal sections of the roots [14,15,20,37,44,45], especially earlier studies using CLSM imaging [13,46,47,48]. Depending on where exactly the samples were split, the penetration depths assessed can vary greatly. Studies assessing the orovestibular plane of longitudinal sections evaluated the “wings” of the butterfly penetration pattern and consequently obtained better values [13,47], while studies assessing the mesiodistal plane [45] only evaluated areas with lesser penetration depths and therefore achieved lower values. A study by Vatkar et al. (2016), for example, evaluated both the orovestibular and mesiodistal planes in the coronal and medial positions, while for the apical position only the mesiodistal plane was evaluated; this may have led to underestimation of the penetration depths in the apical position [13]. Unfortunately, most studies using longitudinal sections have not specified the direction of the split [15,37,46,48]. The butterfly pattern therefore appears to have been largely neglected. Another problem is that different criteria have been used to determine the effectiveness of the disinfection measures. For example, a distinction is often not made between depths at which complete or only some reduction in bacteria was detectable, or no further details are provided on how exactly the “depth of effective disinfection” was defined. Some studies have investigated the depth at which vital bacteria were still found [14], others the depth up to which dead bacteria were detectable [13], and still others have assessed the percentage reduction in bacteria in different dentin layers [46,47]. Dye penetration or discoloration tests, on the other hand, do not allow any conclusions to be drawn about the bacterial reduction achieved at different depths [10,17,18,19,37,38,39,40]. The results obtained are therefore difficult or almost impossible to compare, and further studies using standardized methods and definitions of the “depth of effective disinfection” are needed.

Since in vitro models of any kind can never fully replicate the complexity of biological conditions in vivo, the results of the present study should be interpreted with caution. The use of a mono-species biofilm model as well as the evaluation of only three slices representing the coronal, medial, and apical planes of the root must be considered limitations of the present study. Since additional red background fluorescence must be carefully distinguished from red fluorescence arising from dead bacteria and/or released DNA/RNA, the evaluation and interpretation of the data remains challenging, particularly due to tooth-specific factors and evaluator subjectivity. Nevertheless, the results underline the importance of carefully performed disinfection in endodontic treatment and the need for further studies on the depth of effective disinfection of endodontic disinfection measures. Moreover, further studies are needed to evaluate the influence of patient-specific factors on the depth of effective disinfection in endodontics and to allow for clinical recommendations to translate experimental findings into evidence-based clinical applications.

## 4. Materials and Methods

Tooth preparation, pretreatment and inoculation protocols, and CLSM-guided evaluation were carried out in accordance with the previously evaluated and published methodology [27], applying only pretreatment protocol 2 (Pr2, without NaOCl) and inoculation by overflow (process A) (A-Pr2). The protocol used in the present study is described briefly below.

### 4.1. Tooth Selection

Thirty freshly extracted, caries-free, intact permanent human teeth with straight root canals were selected. Teeth that had previously undergone, or were affected by, crown restoration, root canal treatment, resorption, or incomplete root growth were excluded from the study. All selected teeth were initially sterilized and stored in 0.9% sodium chloride (NaCl), and then underwent crown removal using a diamond saw (Primus cut-grinder; Walter Messner GmbH, Oststeinbek, Germany) with water cooling to obtain a standard root segment 12 mm long.

### 4.2. Preparation and Pretreatment

First, a glide path was prepared with hand files from size 08.02 to size 25.02 (K-files; VDW GmbH, Munich, Germany) with a working length of 11 mm, since the root length was defined as 12 mm patency. Subsequently, mechanical root canal preparation was carried out using rotary files up to size 50.05 (Protaper Gold F1-F5; Dentsply Sirona Inc., Bensheim, Germany). NaCl (at a room temperature of approximately 20 °C) was used for irrigation during instrumentation, at a volume of 1 mL between files. All samples were then subjected to pretreatment protocol Pr2 (Table 2) using a 27-G side-vented needle (Covidien plc, Dublin, Ireland) for irrigation and EDDY (VDW GmbH, Munich, Germany) for sonic activation.

After pretreatment in accordance with protocol Pr2, the root segments were stored overnight in ultrapure water. The samples were then inoculated with *E. faecalis*, except for a few samples that served as negative controls.

### 4.3. Bacterial Inoculation in an Overflow Model

The new overflow model for inoculating teeth (tubules) inside-out with *E. faecalis* was used [27]. Briefly, the roots were placed in sterile 1.5 mL Eppendorf tubes. The gap between the tube and the root was sealed with ultraviolet-curing single-component resin (Eukitt 4400 LB; Walter Messner GmbH, Oststeinbek, Germany) and cured for 20 min using a light polymerization box (Karlo; Walter Messner GmbH, Oststeinbek, Germany). The fixed roots were then sterilized by autoclaving prior to inoculation the following day. It was crucial that the root canals (and dentinal tubules, as far as possible) were dried of any residual water using sterile paper points before inoculation, in order to ensure access of nutrient-rich medium.

Gram-positive *E. faecalis* (strain ATCC 29212) was used as the test organism, which was thawed from frozen stock. After an initial growth phase on brain–heart infusion (BHI) agar plates, one or two colonies were selected for inoculation in 1 mL BHI broth (Oxoid Deutschland GmbH, Wesel, Germany). The resulting suspension was then cultured at 37 °C in aerobic conditions. After 24 h, the suspension had a concentration of 10^9^ colony-forming units (CFUs)/mL, as determined by dilution and plating. Of this *E. faecalis* suspension, 15 µL (comprising approximately 15 million CFUs) and 200 µL of Mueller–Hinton (MH) broth (Becton Dickinson GmbH, Heidelberg, Germany) were then transferred to the previously prepared Eppendorf tubes (containing the root to be inoculated) and incubated for 4 weeks in aerobic conditions at 37 °C. The growth medium was replenished three times a week to ensure optimal growth dynamics.

### 4.4. Grouping and Treatment

After inoculation, the *E. faecalis*-loaded specimens were divided into three groups to examine the effects of different endodontic disinfection measures. The treatment protocols were as follows:

1. Negative control group (*n* = 5): no bacterial inoculation and no treatment (i.e., disinfection measures).
2. Positive control group (*n* = 10): bacterial inoculation and no treatment (i.e., disinfection measures).
3. Conventional irrigation group (*n* = 10): bacterial inoculation and treatment (i.e., disinfection measures). Samples were irrigated using a 27-G side-vented needle (Covidien, Dublin, Ireland) with 2 mL ethylenediamine tetraacetic acid (EDTA) (17%) (SPEIKO^®^—Dr. Speier GmbH, Bielefeld, Germany) for 60 s, followed by 5 mL NaOCl (3%) (SPEIKO^®^—Dr. Speier GmbH, Bielefeld, Germany) for 60 s, and finally 5 mL NaCl for 60 s to neutralize NaOCl traces.
4. Sonically activated irrigation group (EDDY) (*n* = 10): bacterial inoculation and treatment (i.e., disinfection measures). The samples were irrigated with 2 mL EDTA (17%) for 60 s using the same type of needle as in (2), followed by 1 mL NaOCl (3%) for 15 s and sonic activation (5000–6000 Hz) with EDDY (VDW GmbH, Munich, Germany) for 30 s. Irrigation with 1 mL NaOCl (3%) for 15 s and sonic activation for 30 s with EDDY was repeated, followed by irrigation with 3 mL NaOCl (3%) for 30 s and finally 5 mL NaCl for 60 s to neutralize NaOCl traces.

To avoid contamination, the irrigation needles and EDDY tips were changed between samples.

### 4.5. Preparation of Samples for Evaluation

After treatment, the roots were cut perpendicular to the longitudinal axis of the tooth at distances of 2, 5, and 8 mm from the root apex using a diamond saw (cut-grinder Primus; Walter Messner GmbH, Oststeinbek, Germany) with water cooling. This resulted in three slices, each 3 mm thick, representing the coronal, medial, and apical planes of the root. The resin layer was removed from the root surface of the slabs. In addition, example longitudinal slices were obtained from additional samples. The coronal side of each sample slice was then polished using silicon carbide grinding disks with grit sizes of 1200, 2400, and 4000 µm (Waterproof Silicon Carbide Paper FEPA, Struers S.A.S., Champigny-sur-Marne, France, used on EXAKT 400CS, EXAKT Advanced Technologies GmbH, Norderstedt, Germany). For LIVE/DEAD staining, the FilmTracer™ LIVE/DEAD Biofilm Viability Kit L10316 (Molecular Probes, Eugene, OR, USA) was used. During staining and transport to the CLSM unit, the specimens were kept in darkness.

### 4.6. Confocal Laser Scanning Microscopy (CLSM)

After staining, the samples were meticulously washed in ultrapure water for 10 s and placed in µ-Slide eight-well chambers (ibidi GmbH, Gräfelfing, Germany). Images were acquired using an inverted Zeiss LSM710 confocal laser scanning microscope (Carl Zeiss AG, Oberkochen, Germany) and Zen black acquisition software (version 2.3 SP1, Carl Zeiss AG, Oberkochen, Germany). Excitation of SYTO 9 and propidium iodide was performed using a 488 nm laser line, while emission was collected with collection windows of either 493–584 nm for SYTO 9 or 604–718 nm for propidium iodide. Overview images of the samples were obtained using a 10× EC Plan-Neofluar objective in 3 × 3, 4 × 4, or 5 × 5 tile scans (depending on sample size), while a 40× LD C-Apochromat water immersion objective was used for detailed close-up images.

### 4.7. Assessment of Sclerosis Grading

As sclerosis of the dentinal tubules is an important factor for reproducible inoculation and has a major impact on the efficiency of disinfection measures, the degree of sclerosis in all of the teeth included in this study was evaluated on the basis of the upper surface of the 12 mm root segment, to ensure equal distribution between groups and to allow for exclusion of inappropriate samples if necessary. After treatment and sectioning of the samples for subsequent analysis, the degree of sclerosis was assessed for all positions (coronal, medial, apical). Specimens with minimal sclerosis without a butterfly pattern were classified as sclerosis grade (SCG) 1. When a distinct butterfly pattern was visible (with sclerosis in the mesiodistal direction), the specimens were classified as SCG 2, while specimens with the most sclerosis (associated with fracture lines) were classified as SCG 3 (Table 3) [27].

### 4.8. Evaluation and Assessment of Treatment

LIVE/DEAD staining allowed for visual identification and differentiation of vital and dead bacteria in the dentin, with vital bacteria showing green fluorescence, while dead bacteria and/or released DNA/RNA additionally showed red fluorescence [49,50,51]. The CLSM images obtained were analyzed using the Zen Lite Blue software (version 3.6.095.02; Carl Zeiss AG, Oberkochen, Germany) for the following parameters:

1. Bacterial colonization (resulting from the inoculation) of the samples with vital bacteria (green fluorescence) was evaluated in order to determine the penetration depth of *E. faecalis* into the dentinal tubules (positive control group).
2. Bacterial eradication resulting from the disinfection measures investigated (red fluorescence) was evaluated in order to assess the penetration depth or, more precisely, the depth of effective disinfection by the disinfection measures (test groups).

**Figure 4:**
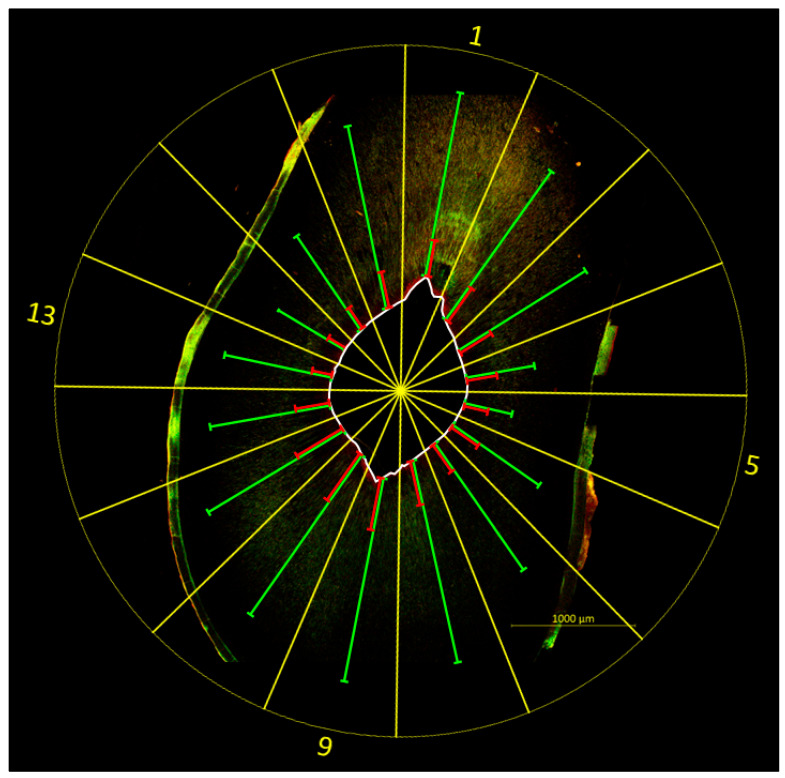
Example image illustrating the evaluation method. A template (yellow) was placed over the image of the tooth sample (sonically activated irrigation (EDDY) group, coronal position, SCG 2), dividing the tooth slice into 16 equal-sized segments (S1–16). The center of the template was aligned with the center of the root canal, with S1 pointing to the oral side of the tooth. The depths of bacterial colonization (green) and bacterial eradication (red) were quantified for each segment by measuring the distance between the root canal wall (white) and the deepest point at which vital bacteria (bacterial colonization) or dead bacteria and/or released DNA/RNA (bacterial eradication) were present. Increased fluorescence is visible on the root surface, originating from root cementum and resin residues.

Both bacterial colonization and bacterial eradication were evaluated on the basis of the individual penetration depths, with the root canal wall serving as reference. For this purpose, a template was placed over the images of the samples, dividing each tooth slice into 16 equally sized segments (S1–16). The center of the template was aligned with the center of the root canal, with S1 pointing to the oral side of the tooth. Bacterial colonization was quantified for each segment by measuring the distance between the root canal wall and the deepest point at which vital bacteria (indicated by green fluorescence) were present in the dentin. Similarly, bacterial eradication was quantified for each segment by measuring the distance between the root canal wall and the deepest point at which dead bacteria or released DNA/RNA (indicated by red fluorescence) were present (Figure 4). Additional red background fluorescence had to be carefully distinguished from red fluorescence arising from dead bacteria and/or released DNA/RNA. This resulted in 16 individual measurement values for bacterial colonization or bacterial eradication per sample, with the mean of the 16 individual measurements per sample being used for further comparison of penetration depths.

### 4.9. Statistical Analysis

Data were analyzed using one-way analysis of variance (ANOVA), followed by the least significant difference test, applying GraphPad Prism version 10 (GraphPad Software, Boston, MA, USA).

## 5. Conclusions

This pilot study confirms our recently published CLSM methodology [27], with high-resolution distinction between vital and dead bacterial cells being a major advantage. Overall, sonically activated irrigation (EDDY) provided a deeper—and more importantly a more homogeneous—radial (omnidirectional) irrigation pattern (less butterfly penetration pattern). In addition, it was confirmed that the irrigation depth depends significantly on the sclerosis grade and position. These results underline the importance of carefully performed disinfection in endodontic treatment and the need for further studies on the depth of effective disinfection of endodontic disinfection measures.

## Figures and Tables

**Table 1:** Heat map for depth of bacterial eradication (mean values in µm) depending on the position and degree of sclerosis for the conventional (conv.) and sonically activated irrigation (EDDY) groups. Values are highlighted using the traffic light method from high (green) to low (red).

			Treatment		Position		
		All	Conv.	EDDY	Coronal	Medial	Apical
All		490					
Treatment	Conv.	450					
	EDDY	520					
Position	Coronal	680	600	760			
	Medial	440	360	520			
	Apical	340	390	280			
Sclerosis grade	1	670	590	760	780	540	550
	2	400	440	360	490	330	400
	3	300	230	360	520	430	200

**Table 2:** Pretreatment protocol Pr2 (adapted from [27]).

	Irrigant/Activation	mL	Time (s)
0.	NaCl between files	1	15
1.	Ultrapure water	5	60
2.	EDTA (17%)	5	60
3.	Activation with EDDY		30
4.	Ultrapure water	5	60

All irrigants were used at a room temperature of ≈20°. EDTA, ethylenediamine tetraacetic acid.

**Table 3:** Sclerosis grading (SCG) of the specimens (ten roots cut into coronal, medial, and apical sections).

	All	SCG 1	SCG 2	SCG 3
Positive control group	30	9	16	5
Conventional irrigation group	30	13	10	7
Sonically activated irrigation group (EDDY)	30	12	10	8

## Data Availability

The data presented in this study are available on request from one of the first authors (R.M.).
